# Anti-α-Glucosidase Activity by a Protease from *Bacillus licheniformis*

**DOI:** 10.3390/molecules24040691

**Published:** 2019-02-15

**Authors:** Chien Thang Doan, Thi Ngoc Tran, Minh Trung Nguyen, Van Bon Nguyen, Anh Dzung Nguyen, San-Lang Wang

**Affiliations:** 1 Department of Chemistry, Tamkang University, New Taipei City 25137, Taiwan; doanthng@gmail.com (C.T.D.); tranngoctnu@gmail.com (T.N.T.); 2 Department of Science and Technology, Tay Nguyen University, Buon Ma Thuot 630000, Vietnam; nguyenminhtrung2389@gmail.com (M.T.N.); bondhtn@gmail.com (V.B.N.); 3 Institute of Biotechnology and Environment, Tay Nguyen University, Buon Ma Thuot 630000, Vietnam; nadzungtaynguyenuni@yahoo.com.vn; 4 Life Science Development Center, Tamkang University, New Taipei City 25137, Taiwan

**Keywords:** anti-α-glucosidase, protease, diabetes, microbial conversion, *Bacillus licheniformis*

## Abstract

Anti-α-glucosidase (AAG) compounds have received great attention due to their potential use in treating diabetes. In this study, *Bacillus licheniformis* TKU004, an isolated bacterial strain from Taiwanese soil, produced AAG activity in the culture supernatant when squid pens were used as the sole carbon/nitrogen (C/N) source. The protein TKU004P, which was isolated from *B. licheniformis* TKU004, showed stronger AAG activity than acarbose, a commercial anti-diabetic drug (IC_50_ = 0.1 mg/mL and 2.02 mg/mL, respectively). The molecular weight of TKU004P, determined by sodium dodecyl sulfate-polyacrylamide gel electrophoresis (SDS-PAGE), was 29 kDa. High-performance liquid chromatography (HPLC) analysis showed that TKU004P may be a protease that demonstrates AAG activity by degrading yeast α-glucosidase. Among the four chitinous sources of C/N, TKU004P produced the highest AAG activity in the culture supernatant when shrimp head powder was used as the sole source (470.66 U/mL). For comparison, 16 proteases, were investigated for AAG activity but TKU004P produced the highest levels. Overall, the findings suggest that TKU004P could have applications in the biochemical and medicinal fields thanks to its ability to control the activity of α-glucosidase.

## 1. Introduction

Alpha-glucosidase (EC 3.2.1.20) is a hydrolytic enzyme that acts mainly on α-1→4 glycosidic linkages of complex carbohydrates to release a single α-glucose [1]. Many living creatures can produce α-glucosidase, including bacteria, yeast, plants and animals [2,3,4,5,6]. Based on the specific substrate, α-glucosidases are separated into three types: types I, II and III. Type I rapidly hydrolyzes aryl glucosides, type II hydrolyzes maltooligosaccharides and type III can hydrolyze both α-glucan and maltooligosaccharides [3]. In humans, α-glucosidase is secreted by the epithelial cells of the small intestine and cleaves dietary carbohydrates into glucose. The release of glucose from complex carbohydrates is the main reason for postprandial hyperglycemia, an important factor in developing type II diabetes [7]. As such, counteracting the activity of α-glucosidase may decrease postprandial hyperglycemia and, as a result, prevent the onset of diabetes [8,9,10].

Anti-α-glucosidase (AAG) compounds are produced via plants, microorganisms and chemical synthesis [7,8,11,12,13,14,15,16,17,18,19,20,21,22,23,24]. Microbial conversion, using bacteria [19,20,21,22,23,24,25,26,27,28,29,30,31,32] or fungi [12,33,34], was suggested as an alternative mean of obtaining natural AAG compounds. The *Bacillus* species in particular has shown potential as a bacterial source, as several strains have been extensively investigated for AAG production, including *B. subtilis* S10 [28], *B. subtilis* B2 [27], *Bacillus* sp. [25] and *B. mycodes* TKU040 [22]. In the previous study, *Bacillus licheniformis* TKU004 demonstrated AAG and protease activities when squid pens were used as the sole carbon/nitrogen (C/N) source during liquid fermentation [20,35], however the production and characterization of AAG from TKU004 strains was not explored. There is currently great interest in the potential of producing AAG via the conversion of fishery by-products by *B. licheniformis* TKU004.

In the current study, a protein which showed strong AAG activity, TKU004P, was extracted from the culture supernatant of TKU004. TKU004P was also explored for the AAG mechanism and as well as the character, whereby it was identified as a protease. To determine potential protease production on medium containing chitinous materials [35], *B. licheniformis* TKU004 was cultured using four fishery by-products as the sole C/N sources: squid pen powder (SPP), demineralized shrimp shell powder (deSSP), shrimp head powder (SHP) and demineralized crab shell powder (deCSP). A comparison of AAG from 16 different proteases was also performed.

## 2. Results and Discussion

### 2.1. Extraction of AAG Protein

**Figure 1:**
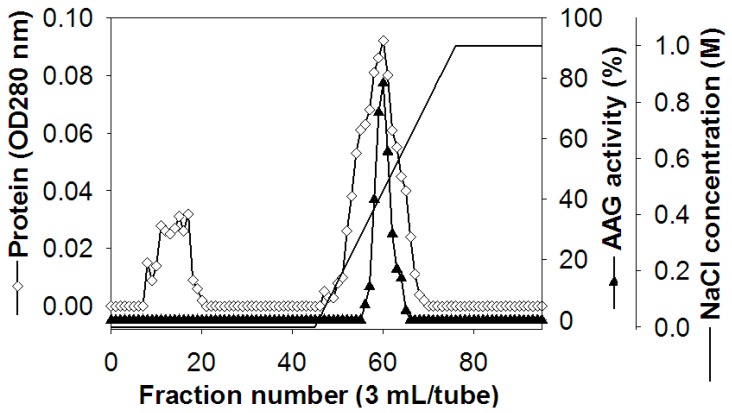
Elution profile of TKU004P on Macro-Prep High S chromatography.

**Figure 2:**
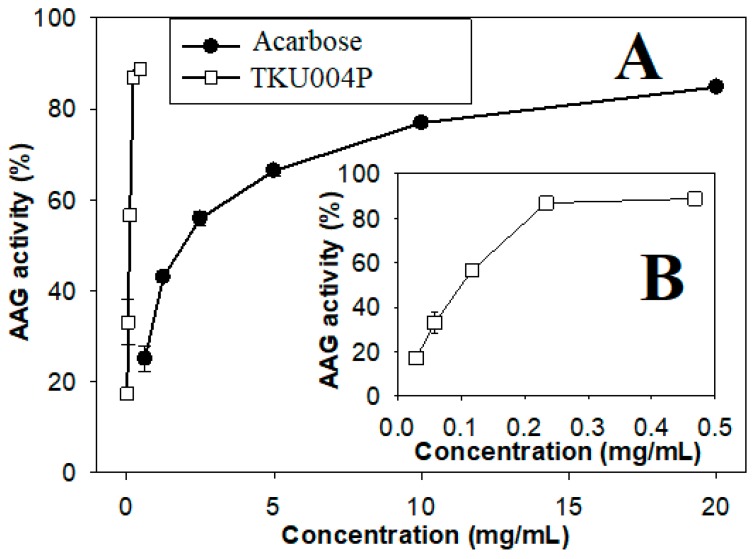
Anti-α-glucosidase (AAG) activity of TKU004P and acarbose. (**A**) Concentration range of 0–20 mg/mL; (**B**) concentration range of 0–0.5 mg/mL.

The AAG protein from TKU004 was isolated and purified by a series of steps. The protein was precipitated from the culture supernatant by ammonium sulfate (80% *w/v*) and eluted by Macro-Prep High S chromatography with a linear gradient of 0–1 M NaCl. The chromatogram showed only one peak of AAG activity (Figure 1). The eluted peak fractions were pooled and desalted for further purification. The second round of elution used Macro-Prep High S chromatography with a linear gradient of 0–0.5 M NaCl. After isolation and purification, approximately 4.04 mg of protein (TKU004P) was obtained. The obtained protein was then tested for AAG activity at a range of concentrations. As shown in Figure 2, TKU004P expressed maximum AAG activity at 0.47 mg/mL with 88.85% and an IC_50_ value of 0.1 mg/mL. Acarbose, a commercial AAG compound, was used as a control; its maximum AAG activity was 84.82% at 20 mg/mL with an IC_50_ value of 2.02 mg/mL. Since TKU004P expressed approximately 20.2-fold stronger AAG activity than acarbose, further investigations were conducted.

**Figure 3:**
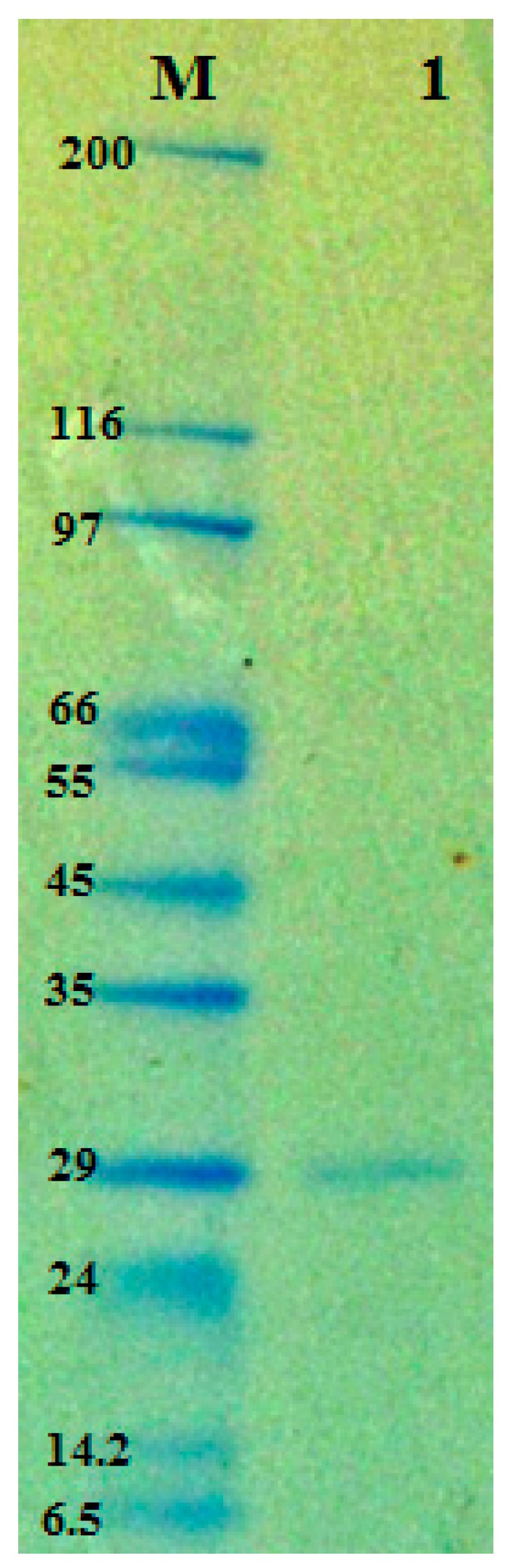
SDS-PAGE analysis of the protease produced by *B. licheniformis* TKU004. M—molecular markers; 1—TKU004P.

The molecular weight of TKU004P was approximately 29 kDa, as determined by the SDS-PAGE method (Figure 3), which was higher than that of other AAG peptic compounds. In previous studies, AAG activity from peptic compounds was rarely reported and most of those were small peptides with molecular weights under 10 kDa [9,36,37].

### 2.2. AAG Mechanism

**Figure 4:**
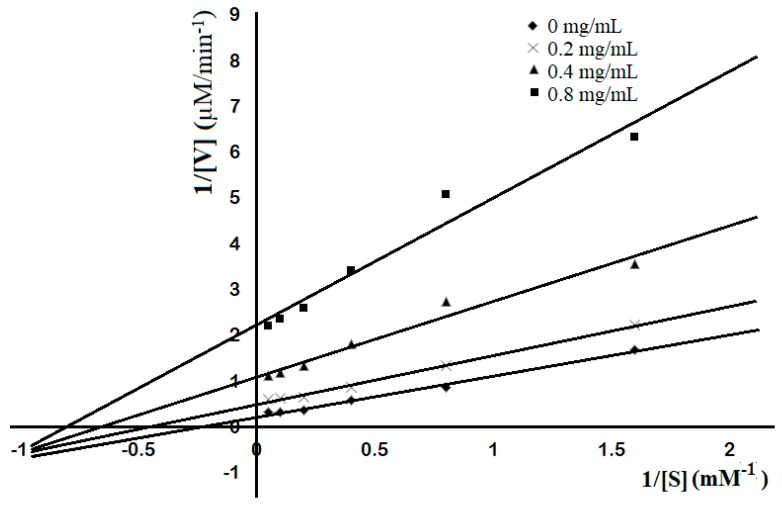
Lineweaver–Burk plot analysis of AAG by TKU004P. *p*-nitrophenyl glucopyranoside (*p*NPG) was used as substrate. The concentration of TKU004P was 0 mg/mL (♦); 0.2 mg/mL (×); 0.4 mg/mL (▲); 0.8 mg/mL (■).

**Figure 5:**
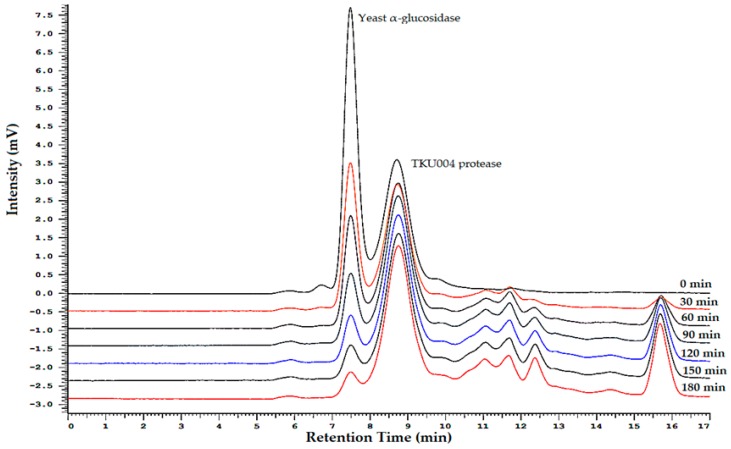
HPLC analysis of the time-course reaction of TKU004P and yeast α-glucosidase.

Initially, a Lineweaver–Burk plot analysis of TKU004P, at concentrations ranging from 0 to 0.8 mg/mL, was used to explore the AAG mechanism. As shown in Figure 4, when the concentration of TKU004P increased, the V_max_ and K_max_ values of α-glucosidase decreased, but not in a similar pattern. Kinetic data revealed that the action of TKU004P was not consistent with enzyme inhibition models like competitive inhibition, non-competitive inhibition or uncompetitive inhibition. Therefore, a time-course HPLC chromatography of the reaction was obtained to analyze TKU004P’s mechanism of action. As shown in Figure 5, there were two main peaks, representing yeast α-glucosidase (with a retention time of 7.5 min) and TKU004P (with a retention time of 8.8 min). At 0 min, the intensity value of yeast α-glucosidase reached 7.3 mV, then dramatically decreased as the reaction time increased. In addition, peaks of small peptides with retention times over 10 min appeared after 30 min of reaction and the area increased over time. According to Table 1, there was a strong relationship between the relative residual concentration and the activity of yeast α-glucosidase, resulting in similar patterns after 30 min with values of 49.90% and 46.06%, respectively. Both values nearly disappeared after 180 min, dropping down to 4.45% and 2.33%, respectively. It was suggested that yeast α-glucosidase was degraded by TKU004P to form decomposition products, thereby reducing its activity. Therefore, the AAG activity of TKU004P could be achieved by a proteolytic mechanism, in which TKU004P acted as the protease and glucosidase was the substrate.

Due to the close relationship between diabetes mellitus and the action of α-glucosidase in the small intestine, the screening of AAG compounds has received much attention by researchers in recent years. Unlike other studies, in which selected compounds served as α-glucosidase inhibitors [19,20,21,22,23,24,25,26,27,28,29,30,31,32], the isolated protein from TKU004 showed AAG activity by degrading the enzyme. As such, this work could be a novel contribution to AAG research.

### 2.3. Proteolytic Activity

Since TKU004P showed ability in degrading yeast α-glucosidase, this protein was considered as a protease. Compared to our previous study, only one metalloprotease (M_w_ = 27 kDa) was isolated from *B. licheniformis* TKU004 on SPP medium [34]. TKU004P, due to its similar molecular weight and production conditions, could be that metalloprotease; however, the activity of TKU004 metalloprotease on different substrates has not been extensively studied. Therefore, numerous protease substrates were used to explore the proteolytic activity of TKU004P. As shown in Table 2, TKU004P displayed diminishing proteolytic activity in the order of casein > fibrinogen > hemoglobin; low or non-activity was seen on gelatin, albumin, elastin and myoglobin. By using a modified protein as the substrate, TKU004P showed the most activity on azocasein, followed by azoalbumin. These results indicate that TKU004P is a caseinolytic protease. When testing protease activity on chromogenic substrates, TKU004P showed the most activity on *N*-succinyl-Ala-Ala-Pro-Phe-*p*-nitroanilide, the specific substrate of chymotrypsin/subtilisin. It had a lesser effect on *N*-benzoyl-Val-Gly-Arg- *p*-nitroanilide and d-Val-Leu-Lys-*p*-nitroanilide, the specific substrate of plasmin (Table 2). According to other reports, numerous proteases from *Bacillus* species were classified at subtilisin-like, for instance *B. licheniformis* [38], *B subtilis* [38], *B. amyloliquefaciens* [39], *B. intermedius* [40] and *B. pumilus* [41]. As such, TKU004P was considered to be a subtilisin-like protease with caseinolytic activity.

### 2.4. pH Stability of TKU004P

**Figure 6:**
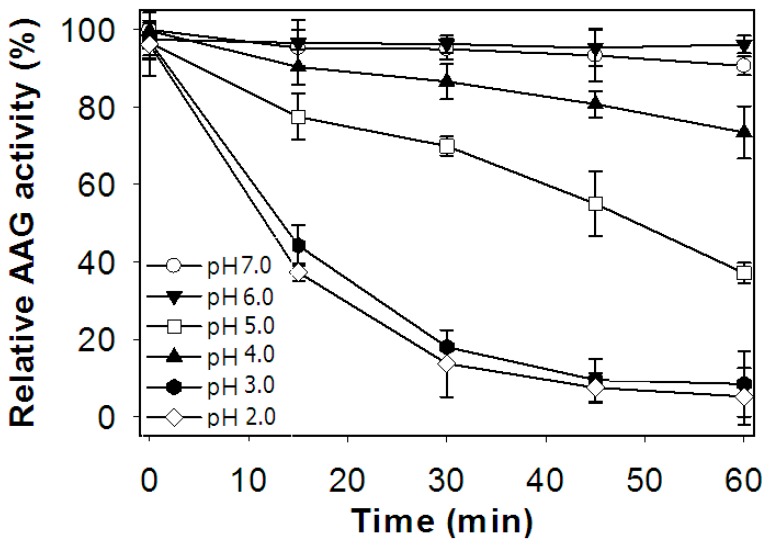
Effect of pH on the stability of TKU004P.

To determine its pH stability, TKU004P was treated with a range of pH values at 37 °C for 60 min; the residual AAG activity was analyzed at pH 7.0. According to Figure 6, the AAG activity of TKU004P was stable at pH 6.0–7.0, with over 90% of its original activity retained. At pH 5.0, TKU004P maintained 86.60% and 73.46% of its initial activity after 30 min and 60 min of treatment, respectively. At pH 4.0, TKU004P showed passable stability after 30 min of incubation with 69.95% activity remaining. However, at pH 2.0–3.0 TKU004P lost over 50% of its ability after 15 min of incubation and lost 90% after 60 min of incubation. These results indicate that TKU004P is not stable in extremely acidic conditions. Therefore, in order to maintain function in the intestinal tract, it should be taken into account that the stomach pH is very acidic and could strongly influence the AAG activity of TKU004P.

### 2.5. Utilization of C/N Source for TKU004P Production

**Figure 7:**
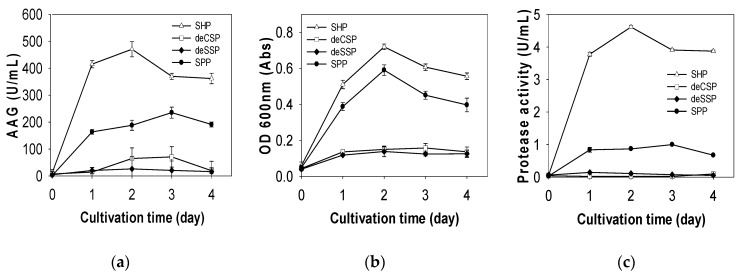
Effect of the C/N source on AAG (**a**), optical density (OD) (**b**) and protease activity production (**c**) of *B. licheniformis* TKU004.

To determine the best C/N source for TKU004P production, the strain was incubated in a liquid medium containing 1% (*w/v*) of different fishery by-products, including SPP, SHP, deCSP and deSSP. As shown in Figure 7, AAG activity was highest using SHP (470.66 U/mL, 2 days). In the previous report, SPP was a better C/N source than deCSP (174.59 U/mL and 91.29 U/mL, respectively) [20]. In the current study, AAG activity for SPP (235.28 U/mL, 3 days) was higher than that for both deCSP (70.97 U/mL, 3 days) and deSSP (26.37 U/mL, 2 days).

Similar to the results for AAG activity, protease activity was higher with SHP (4.61 U/mL, 2 days) than SPP (0.99 U/mL, 3 days), deSSP (0.14 U/mL, 1 day) or deCSP (0.09 U/mL, 4 days) (Figure 7). Of the various C/N sources, including SSP, SCSP, KM, KB, CFSS and CFCS, SPP is reported as the best source for producing protease by TKU004 [34]. As such, using SHP as the sole C/N source could result in higher protease levels than using SPP. 

The relationship between anti-glucosidase, protease activity and bacterial growth (optical density (OD) 600 nm) was studied herein. As shown in Figure 7, SHP was the best C/N source for cell growth (0.72 Abs, 2 days), producing better results than SPP (0.59 Abs, 2 days), deCSP (0.16 Abs, 3 days) or deSSP (0.14 Abs, 2 days). Based on the similar results, one can assume there is a direct relationship between anti-glucosidase, protease activity and bacterial growth.

Shrimp heads are one of the by-products of fish processing, therefore the conversion of this material via microorganisms to produce bioactive compounds could lower overall costs. In fact, SHP has been reported to produce bioactive compounds in several studies; for instance: chitosanase from *B. cereus* TKU027 [42], α-glucosidase inhibitor from *Staphylococcus* sp. TKU043 [21], nattokinase from *B. subtilis* TKU007 [43] and protease from *B. cereus* TKU022 [44]. Therefore, there is great interest in recycling SHP to produce TKU004P by *B. licheniformis* TKU004.

### 2.6. Effect of TKU004P on Different Enzymes

**Figure 8:**
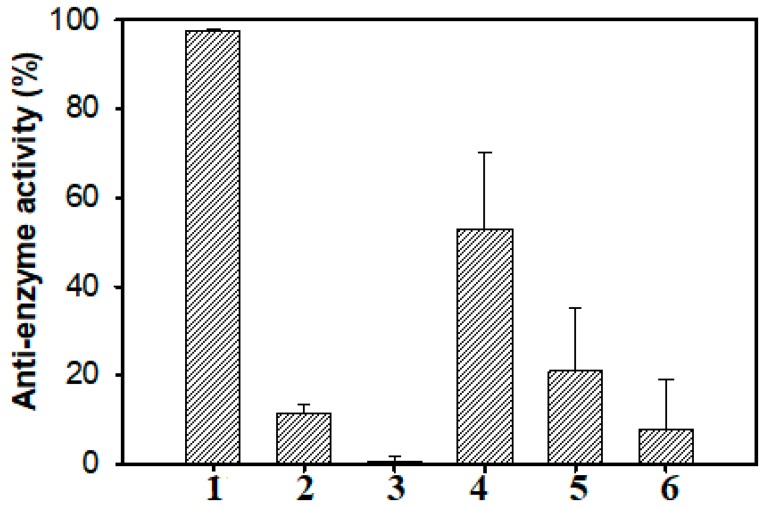
Effect of TKU004P on different enzymes. **1**—yeast α-glucosidase; **2**—rat α-glucosidase; **3**—porcine pancreatic α-amylase; **4**—*B*. *subtilis* α-amylase; **5**—lysozyme; **6**—cellulose.

Six commercial enzymes were used to test the anti-enzyme activity of TKU004P. These included yeast α-glucosidase, rat α-glucosidase, porcine pancreatic α-amylase, *B. subtilis* α-amylase, lysozyme and cellulase. As shown in Figure 8, TKU004P showed strong activity against yeast α-glucosidase (97.65%) but had a weaker or non-response against porcine pancreatic α-amylase (53.07%), *B. subtilis* amylase (20.91%), rat α-glucosidase (11.56%), cellulase (7.95%) and lysozyme (0.74%). These results indicate that TKU004P specifically counteracted the action of yeast α-glucosidase. As such, TKU004P may have potential use in biochemical or medicinal fields thanks to its ability to control the activity of α-glucosidase.

### 2.7. Comparison of AAG Activity by Different Proteases

Since TKU004P showed strong AAG activity, it was compared against the activity of other proteases. In this study, proteases from 14 bacterial strains isolated from Taiwan soils and two plants (papain and bromelain) were tested for AAG activity. Of these, TKU004 protease showed the highest AAG activity with 98.38% against yeast α-glucosidase, while less than 40% of AAG activity was due to other proteases (Table 3). In the previous report, culture supernatant of *B. licheniformis* TKU004 showed strong AAG activity along with those of *Paenibacillus* strains, including *Paenibacillus* sp. TKU042, a bacterial strain extensively studied in anti-diabetes research [20]. By comparing the results in the current study, it was indicated that only TKU004 protease showed an efficient effect on the action of yeast α-glucosidase.

TKU004P has been reported to have potential use in chitin extraction. Moreover, TKU004 has shown valuable characteristics, such as solvent stability as well as the ability to be produced on medium containing SPP, a by-product of fishery processing [35]. In the current study, for the first time TKU004P demonstrated AAG activity by degrading α-glucosidase. The results also showed that TKU004P possessed the strongest AAG activity among all those tested, suggesting that it may be a good candidate for AAG.

## 3. Materials and Methods 

### 3.1. Materials

Squid pens, crab shells and shrimp shells were obtained from Shin-Ma Frozen Food Co. (I-Lan, Taiwan) [45]. Shrimp head powder (SHP) was acquired from Fwu-Sow Industry (Taichun, Taiwan). Demineralized shrimp shell powder (deSSP) and demineralized crab shell powder (deCSP) were prepared according to the previously described methods [45]. Acarbose, *p*-nitrophenyl glucopyranoside and enzymes (yeast α-glucosidase, rat α-glucosidase, porcine pancreatic α-amylase, *B. subtilis* α-amylase, lysozyme, cellulase, bromelain and papain) were obtained from Sigma Chemical Co., St. Louis, MO, USA. The Macro-Prep High S column was obtained from BioRad (Hercules, CA, USA). All other reagents used were of the highest grade available.

### 3.2. AAG Activity

The AAG assay was modified from the methods of Nguyen et al. (2017) [23]. A mixture of 50 µL of the sample, 50 µL of α-glucosidase solution (1 U) and 100 µL of Tris-HCl buffer was kept at 37 °C for 30 min to allow the sample to counteract the enzyme. Then, 50 µL of *p*NPG was then added to the mixture, which was maintained at 37 °C for 30 min. To stop the reaction, 100 µL of 1 M Na_2_CO_3_ solution was added. The mixture was then measured for optical density (OD) at 410 nm using an ELISA absorbance reader (Bio-Rad, USA). The AAG activity was calculated using the following formula:

[AAG (%) = (A − B)/A × 100]

where A is the OD of the solution without the sample, and B is the OD of the solution with the sample. AAG activity was expressed as U/mL and as an IC_50_ value. The volume of a sample that could counteract 50% of enzymatic activity was defined as 1 U, while the IC_50_ value was defined as the concentration of sample that counteracted 50% of enzyme activity under assay conditions. 

### 3.3. Protease Activity Assay

To measure protease activity, 0.1 mL of the sample was mixed with 0.1 mL of casein solution (1% *w/v* in 50 mM Tris-HCl buffer) and incubated for 30 min at 37 °C. The reaction was stopped by the addition of 0.6 mL trichloroacetic acid solution (5%). The methods of Todd were used to measure the activity of the reaction; tyrosine was used as a reference [20]. Protease activity was defined as the amount of enzyme required to release 1 µmol of tyrosine per min.

### 3.4. Extraction of TKU004P

Five hundred milliliters of the cell culture supernatant of *B. licheniformis* TKU004 was concentrated with 80% saturated ammonium sulfate and kept at 4 °C overnight for precipitation. The precipitate was collected by centrifugation at 12,000× *g* for 30 min, then dissolved in 20 mM Tris-HCl buffer (pH 7.0) and dialyzed against 2 L of the same buffer for 48 h at 4 °C. The solution was charged onto a Macro-Prep High S column that had been pre-equilibrated with the same Tris-HCl buffer and eluted with 0–1 M NaCl gradient-containing buffer. The obtained AAG activity fractions were further desalted by dialysis and purification by ion exchange chromatography on the Macro-Prep High S column with 0–0.5 M NaCl gradient-containing buffer. The molecular weight of the sample was determined using the SDS-PAGE method [20].

### 3.5. AAG Mechanism

The sample was prepared at different concentrations (0 mg, 0.2 mg, 0.4 mg and 0.8 mg) in Tris-HCl buffer and added to the α-glucosidase solution following the conditions of the AAG activity assay as described above. Then, 50 µL of *p*NPG at different concentrations (0.625 mM to 20 mM) was added to the mixture. After incubation, the optical density values of the final solutions were obtained at 410 nm and the reaction kinetics were investigated using the Lineweaver–Burk plot analysis [10].

HPLC analysis was also used to test the AAG mechanism. A mixture (1 mL) of the sample and α-glucosidase was incubated at 37 °C for 180 min. Every 30 min, 100 µL was collected for further measurements, including HPLC analysis and AAG activity. HPLC analysis was performed on a Hitachi Chromaster system (Column, KW802.5; mobile phase, 0.3 M NaCl in 20 mM Tris buffer (pH 7.0; sample volume, 20 µL; flow rate, 1.0 mL/min; detector, UV 280 nm, Taipei, Taiwan).

### 3.6. Proteolytic Activity

The sample was incubated with various substrates at 37 °C for 30 min. The protease activity in casein was used as a control to measure the relative activity in other substrates. Under the experimental conditions, a measurement at 440 nm was used to calculate protease activity against azocasein and azoalbumin, and at 410 nm for the chromogenic substrates. The absorbance readings were recorded by using a UV-Vis spectrometer (UV 2550; Shimadzu, Tokyo, Japan)

### 3.7. pH Stability

To determine the effect of pH on enzyme stability, the enzyme was incubated at 37 °C in buffers of different pH levels for 60 min; the residual activity was measured at pH 7.0 every 15 min. The buffer systems used included glycine HCl (50 mM, pH 2.0–3.0), acetate (50 mM, pH 4.0–5.0) and Tris-HCl (50 mM, pH 6.0–7.0).

### 3.8. Utilization of C/N Source for TKU004P Production

Four chitin-containing materials, deCSP, SPP, SHP and deSSP, were used as the sole C/N sources at a concentration of 1% (*w/v*). *B. licheniformis* TKU004 was grown in 100 mL of liquid medium in 250-mL Erlenmeyer flasks containing 1% of each chitinous material, 0.1% K_2_HPO_4_ and 0.05% MgSO_4_·7H_2_O [20]. Seed culture (1 mL) was transferred into the medium and incubated for 4 days at 37 °C on a shaking incubator (150 rpm). Every 24 h, the culture broth was collected for further measurement.

### 3.9. Effect of TKU004P on Different Enzymes

Six enzymes were used to test TKU004′s effect on their activity, including yeast α-glucosidase, rat α-glucosidase, porcine pancreatic α-amylase, *B. subtilis* α-amylase, lysozyme and cellulose. The anti-enzyme assay was modified from the method of Nguyen et al. [24]. In brief, 50 µL TKU004P was added to 50 µL of each enzyme and 100 µL potassium phosphate buffer and kept at 37 °C for 30 min. Subsequently, 50 µL of the substrate was then added to the mixture which was kept at 37 °C for 30 min. The substrate for yeast α-glucosidase and rat α-glucosidase was *p*NPG. Soluble starch was the substrate for porcine pancreatic α-amylase and *B. subtilis* α-amylase, cellulose powder was the substrate for cellulose and *Micrococcus luteus* was the substrate for lysozyme. For yeast α-glucosidase and rat α-glucosidase, the reactions were stopped by adding 100 µL of 1 M Na_2_CO_3_ and the final solution was measured at 410 nm. Meanwhile, 300 µL DNS reagent solution was used to stop the reactions of porcine pancreatic α-amylase, *B. subtilis* α-amylase and cellulose; measurements were performed at 515 nm after heating the final mixtures for 10 min at 100 °C. An OD of 600 nm was used to measure the activity of lysozyme. The effect of TKU004P on the enzymes was expressed as anti-enzyme activity (%) and was calculated by a formula similar to that of AAG.

### 3.10. Comparison of AAG Activity by Different Proteases

Sixteen proteases from bacteria and plants were used to test AGG activity against yeast α-glucosidase, following the AAG assay as described above.

## 4. Conclusions

In this study, TKU004P, an isolated protein from *B. licheniformis* TKU004, possessed stronger AAG than acarbose, a commercial anti-diabetic drug. Unlike other AAG compounds which act like enzyme inhibitors, TKU004P counteracted α-glucosidase via a degrading mechanism. To the best of our knowledge, this is the first report describing AAG ability from a protease. Furthermore, TKU004P not only expressed specific activity against yeast α-glucosidase, it demonstrated the highest activity among the 16 tested proteases, indicating its potential use in counteracting α-glucosidase. Finally, TKU004P production was the highest using shrimp heads, a fishery by-product containing chitin, as the sole C/N source. This suggests that shrimp heads are a viable source for the production of TKU004P via bioconversion by *B. licheniformis* TKU004.

## Figures and Tables

**Table 1:** Result of time-course reaction of TKU004P and yeast α-glucosidase.

Reaction Time (min)	Relative Concentration of α-Glucosidase (%)	Relative Activity of α-Glucosidase (%)
0	100.00	100.00 ± 3.87
30	49.90	46.06 ± 3.61
60	38.68	29.87 ± 3.43
90	21.95	21.84 ± 3.95
120	15.02	4.53 ± 1.42
150	10.20	2.86 ± 0.82
180	4.45	2.33 ± 0.53

**Table 2:** Substrate specificity of TKU004P.

Substrate	Relative Activity (%)		
	Method 1 ^a^	Method 2 ^b^	Method 3 ^c^
Casein (C)	100.00 ± 5.77		
Albumin	0		
Gelatin	3.89 ± 2.77		
Elastin	0		
Hemoglobin	18.40 ± 3.70		
Myoglobin	6.29 ± 0.86		
Fibrinogen	22.11 ± 2.58		
Azocasein (C)		100.00± 4.68	
Azoalbumin		86.60 ± 7.28	
d-val-leu-lys *p*-nitroanilide			26.48 ± 9.04
*N*-benzoyl-val-gly-arg *p*-nitroanilide			7.17 ± 0.97
*N*-succinyl-ala-ala-pro-phe *p*-nitroanilide (C)			100.00 ± 1.11

Note: ^a^, the activities of these substrate were determined by the method of Todd; ^b^, the activities of these substrates were determined by measuring the absorbance at 420 nm; ^c^, the activities of these substrates were determined by measuring the absorbance at 405 nm.

**Table 3:** Comparison of the AAG activity produced by various proteases.

Source of Enzyme	AAG Activity (%)
*B. licheniformis* TKU004	98.38 ± 0.19
*B. subtilis* TKU007	34.63 ± 2.37
*Lactobacillus paracasei* TKU010	2.55 ± 6.83
*Serratia marcescens* TKU011	13.06 ± 3.58
*Serratia ureilytica* TKU013	16.83 ± 4.19
*Pseudomonas tamsuii* TKU015	3.55 ± 5.63
*Serratia* sp. TKU016	6.82 ± 8.26
*Serratia* sp. TKU020	21.58 ± 5.51
*B. mycodes* TKU038	32.75 ± 4.41
*Paenibacillus macerans* TKU029	4.81 ± 3.29
*Paenibacillus mucilaginousus* TKU032	5.72 ± 6.43
*Paenibacillus* sp. TKU042	13.58 ± 5.84
*B. mycodes* TKU040	10.26 ± 7.58
*B. cereus* TKU028	29.44 ± 4.72
Papain	23.51 ± 5.13
Bromelain	26.82 ± 4.68
